# Compressed Sensing Photoacoustic Imaging Reconstruction Using Elastic Net Approach

**DOI:** 10.1155/2022/7877049

**Published:** 2022-12-20

**Authors:** Xueyan Liu, Shuo Dai, Mengyu Wang, Yining Zhang

**Affiliations:** School of Mathematical Sciences, Liaocheng University, Shandong 252000, China

## Abstract

Photoacoustic imaging involves reconstructing an estimation of the absorbed energy density distribution from measured ultrasound data. The reconstruction task based on incomplete and noisy experimental data is usually an ill-posed problem that requires regularization to obtain meaningful solutions. The purpose of the work is to propose an elastic network (EN) model to improve the quality of reconstructed photoacoustic images. To evaluate the performance of the proposed method, a series of numerical simulations and tissue-mimicking phantom experiments are performed. The experiment results indicate that, compared with the *L*_1_-norm and *L*_2_-normbased regularization methods with different numerical phantoms, Gaussian noise of 10-50 dB, and different regularization parameters, the EN method with *α* = 0.5 has better image quality, calculation speed, and antinoise ability.

## 1. Introduction

During the recent 20 years, photoacoustic imaging (PAI) has received wide spread attention because it combines the advantages of both optical and ultrasound imaging [1–3]. PAI can obtain the quantitative information of the light absorption coefficient of biological tissue and hence has been successfully used in various clinical applications, including breast imaging [4, 5], skin imaging [6, 7], cardiovascular imaging [8], and endoscopy [9, 10]. Photoacoustic effect is excited when a short pulse laser irradiates the biological tissue. And the optically absorbing targets in the tissue will produce the photoacoustic signals, which can be received by the ultrasound transducers placed around the tissue. These acquired signals can be utilized to calculate the spatial light absorption distribution inside the tissue through a certain image reconstruction method [11, 12].

The reconstruction algorithm has an important effect on image quality. Traditional reconstruction algorithms, such as FBP and delay and sum algorithms [11–13], are widely used because of their accuracy and convenience. However, these algorithms need to collect complete data to reconstruct a high-quality image. Furthermore, only a limited range of photoacoustic signals can only be obtained in many experiments [14–16]. In this case, the PAI images reconstructed by the traditional algorithms usually suffer from streaking artifacts and edge blurring. Generally, PAI reconstruction with incomplete data is an ill-posed problem, and regularization techniques must be applied to suppress noise and artifacts in the reconstructed images. By adding some prior knowledge or reasonable regularization constraints, the model-based iterative reconstruction algorithms that can further improve the image quality have been developed for PAI [17–19]. One of the algorithms is based on *L*_*p*_-norm regularization, where the *L*_*p*_-norm of the light absorption distribution is applied to constrain the PAI reconstruction. The most popular *L*_2_-norm regularization approach that can achieve better prediction performance has been used in PAI [20, 21]. However, *L*_2_-norm regularization often generates oversmooth solutions. As an alternative, *L*_1_-norm regularization attracted enormous research interest in PAI due to its sparsity-inducing property [22–24]. Nevertheless, the *L*_1_-norm regularization cannot reconstruct an image with the least signals when applied to compress sensing. To be able to achieve more sparse results, the nonconvex *L*_*p*_-norm (0 ≤ *p* < 1) regularizations for PAI have been investigated, which provides better reconstruction results over *L*_1_-norm regularizations [25–27]. The above studies have confirmed that *L*_*p*_-norm regularization has been widely applied in PAI. However, the *L*_*p*_-norm regularization is a nonconvex optimization problem, which is difficult to solve quickly and efficiently.

Recently, the elastic net (EN) regularization is developed for solving the ill-posed inverse problem of *L*_*p*_-norm regularization [28]. As a combination and compromise between *L*_1_- and *L*_2_-norm regularizations, the EN method is useful when the total number of image pixels is much larger than the number of observed signals, which has been applied to many biomedical imaging fields. For example, Majumdar and Ward have used EN to reconstruct MRI images based on the undersampling sparse *k*-space data [29]. Wang et al. applied the adaptive parameter search EN algorithm to calculate the inverse problem of the fluorescence molecular tomography [30]. Causin et al. investigated the application of EN regularization technique in diffuse optical tomography [31]. All the above papers have verified that EN regularization technique can produce a sparse model with good prediction accuracy.

In this paper, the EN regularization is used to optimize the PAI image reconstruction problem. The paper is organized as follows. The photoacoustic theory and the reconstruction algorithm based on the EN regularization are presented in the second section. The third section introduces the experimental processes and results of the numerical simulation and the tissue-mimicking phantom. In Section 4, some conclusions are given.

## 2. Theory and Method

### 2.1. Photoacoustic Theory

According to the photoacoustic signal generation theory, the relationship between pressure *p*(*r*, *t*) at position *r* and time *t* and the laser energy deposition *A*(*r*) in a homogeneous acoustic medium can be described by the following:

(1) $$\begin{array}{c} \left(\nabla^{2}-\frac{1}{c^{2}}\frac{\partial^{2}}{\partial t^{2}}\right)p\left(r,t\right)=-\frac{\beta A\left(r\right)}{C_{p}}\frac{d\delta \left(t\right)}{dt}, \end{array}$$

where *c* is the acoustic speed in the medium; *C*_*p*_ and *β* denote the specific heat and the thermal coefficient of volume expansion; and *δ*(*t*) represents the shape of the laser pulse that can be expressed as a delta function. Based on Green's function, the solution to the time domain forward problem can be expressed as [1]

(2) $$\begin{array}{c} p\left(r_{0},t\right)=\frac{\beta }{4\pi C_{p}}\frac{\partial }{\partial t}{∭}_{\left|\text{r}-r_{0}\right|=ct}\frac{A\left(r\right)}{t}{\text{d}}^{3}r, \end{array}$$

where *r*_0_ is the location of the ultrasonic detector. By denoting *k* = *ω*/*c* and taking the Fourier transform of time *t*, it is easy to obtain the frequency spectrum of the photoacoustic pressure

(3) $$\begin{array}{c} \bar{p}\left(r_{0},k\right)=\frac{-i\omega \beta }{4\pi C_{p}}∭A\left(r\right)\frac{\exp\left(ik\left|r_{0}-r\right|\right)}{\left|r_{0}-r\right|}d^{3}r. \end{array}$$

The purpose of the inverse PAI problem is to obtain an approximation of the absorption distribution *A*(*r*) from a set of sampled data $\bar{p}\left(r_{0},k\right)$. The forward problem can be described using the following matrix relation:

(4) $$\begin{array}{c} Y=KX+e,{\left\|e\right\|}_{2}\leq \varepsilon , \end{array}$$

where *Y* = (*y*_1_, *y*_2_,⋯*y*_*M*_)^*T*^ is a column vector representing the acoustic pressure $\bar{p}\left(r_{0},k\right)$ and *M* is the number of measurements; *X* = (*x*_1_, *x*_2_,⋯*x*_*N*_)^*T*^ denotes the vector of the unknown reconstruction image *A*(*r*) and *N* denotes the number of pixels; and *e* represents the noise. **K** is the projection matrix in the temporal frequency domain with the size of *M* × *N* that can be can be discretized as

(5) $$\begin{array}{c} K_{\left(m,n\right)\left(i,j\right)}={ick}_{n}\frac{e^{-{ik}_{n}\left|r_{m}-r_{ij}\right|}}{\left|r_{m}-r_{ij}\right|}g_{n},m=1,2,\cdots ,p,n=1,2,\cdots ,q, \end{array}$$

where *r*_*m*_ is the position of the ultrasonic transducer and *p* is the total number of location, *n* represents the frequency and *q* is the number of samples, and *r*_*ij*_ indicates the image pixel coordinates, respectively. Generally speaking, the well-known FBP algorithm can obtain better reconstruction accuracy when the signal is sufficient. However, the FBP algorithm has poor prediction accuracy when the data is incomplete, and the regularization methods are needed to improve the reconstruction quality.

### 2.2. *L*_*p*_-Norm Minimization-Based Iterative Reconstruction Algorithm

The PAI image reconstruction with insufficient measurements is essentially an ill-posed inverse source problem. It is difficult for the analytic reconstruction algorithm to reconstruct high-quality images in limited data settings. Therefore, regularization is employed to suppress noise and artifacts and yields an acceptable image. The *L*_2_-norm-based Tikhonov regularization method is often employed to solve the ill-posed problem [21, 32]. We can get the regularized solution of Equation (4) in the framework of Tikhonov by minimizing the following functions:

(6) $$\begin{array}{c} \underset{X}{\min}\frac{1}{2}{\left\|Y-\text{KX}\right\|}_{2}^{2}+\lambda {\left\|\text{LX}\right\|}_{2}^{2}, \end{array}$$

where *λ* is a regularization parameter. And the regularization operator *L* is usually chosen to be the identity matrix or finite difference operators. The Tikhonov regularization can be solved effectively by LSQR algorithm. However, the oversmoothness of the Tikhonov regularization solution will lead to the loss of details in the reconstructed image.

The compressed sensing (CS) theory suggests that an image can be reconstructed exactly from insufficient measurements if it is sparse or can be sparsely represented in an appropriate basis. Fortunately, most of the medical image can be sparsely represented under a suitable sparse transform basis *X* = *Φ*^−1^*θ* , in which *θ* is the sparse transform coefficient. It has been proved that the PAI image is sparse in a discrete wavelet basis and numerical derivative basis [22]. In this paper, *Φ* is defined as four-level symmetric wavelet transform. In order to reconstruct PAI images with insufficient measurements by using the theory of compressed sensing, the following *L*_1_-norm optimization problem can be solved:

(7) $$\begin{array}{c} \underset{X}{\min}\frac{1}{2}{\left\|Y-\text{KX}\right\|}_{2}^{2}+\lambda {\left\|\Phi X\right\|}_{1}. \end{array}$$

At present, the most common solution of Equation (7) is to employ the CS techniques, such as L1-MAGIC [22], YALL1 [23], and SPGL1 [24]. However, *L*_1_-norm-based LASSO regularization usually has some serious flaws such as oversparseness. In recent years, Zou and Hastie have proposed the elastic network regularization methods to improve the problem that the traditional regularization methods are too smooth or too sparse [28].

The functional interpretation of the solution with elastic net regularization term is shown below. Aiming at the problem that the traditional regularization method is oversmooth or oversparse, the elastic network regularization method was proposed. The framework is as follows:

(8) $$\begin{array}{c} \underset{X}{\min}\;\left\{\frac{1}{2}{\left\|Y-\text{KX}\right\|}_{2}^{2}+\lambda \left[\alpha {\left\|\Phi X\right\|}_{1}+\frac{\left(1-\alpha \right)}{2}{\left\|X\right\|}_{2}^{2}\right]\right\}, \end{array}$$

where *λ* is the overall regularization parameter, *α* ∈ [0, 1] denotes the convex combination weight, which decides the weights of the *L*_1_- and *L*_2_-norm terms. *α*‖*ΦX*‖_1_ + ((1 − *α*)/2)‖*X*‖_2_^2^ is the EN penalty, which is a compromise between LASSO and the Tikhonov regularization. This penalty possesses the advantages of both regularizations and meets properly the requirement of image sparsity and smoothness. If *α* = 1, Equation (8) becomes the *L*_1_-norm-based LASSO regularization; If *α* = 0, Equation (8) will become the *L*_2_-norm-based Tikhonov regularization. The quality of image reconstruction by the EN method is usually related to the selection of regularization parameters. It is very important to choose the values of parameters *α* and *λ* correctly.

### 2.3. The Evalution Factors

To quantitatively evaluate the efficiency and accuracy of PAI reconstruction, the CPU running time, the normalized mean absolute error (NMAE), and the peak signal-to-noise ratio (PSNR) were used as quantitative factors. The CPU runtime is used to evaluate the computational efficiency of the PAI reconstruction algorithm. The PSNR is applied to estimate the image quality, and the NMAE is employed to quantify the reconstruction error. The PSNR can be defined by

(9) $$\begin{array}{c} \text{PSNR}\left(\overset{⌢}{X}\right)=10*\log_{10}\left(\frac{N^{*}\max\left(X\right)}{{\left\|X-\overset{⌢}{X}\right\|}^{2}}\right), \end{array}$$

where $\overset{⌢}{X}$ is the reconstruction image and max(*X*) means the maximum value of pixels, which in our simulation is 1. The NMAE is defined as

(10) $$\begin{array}{c} \text{NMAE}\left(\overset{⌢}{X}\right)=\frac{\left\|X-\overset{⌢}{X}\right\|}{\left\|X\right\|\times 100\%}. \end{array}$$

## 3. Experiment and Result

In this section, multiple numerical simulations and applications were performed to validate the effectiveness of the EN method. And the *L*_2_-norm-based Tikhonov regularization method [33] and the *L*_1_-norm-based regularization method SPGL1 [34] are used to compare with the EN method, where the Tikhonov regularization method is a special case of the EN method corresponding to *α* = 0. Both forward projection and backward reconstruction are performed in 2D, where the object to be imaged is approximately contained in a thin plate. The sparse transform operator is set to four-level symmetric wavelet transform using the Rice Wavelet Toolbox. All the MATLAB programs are performed on a desktop computer with a 3.6 GHz CPU and 32 GB memory. In this paper, we use the MATLAB package glmnet to select the appropriate parameters of the EN [35].

### 3.1. Reconstruction from Simulated Sparse-View Data

**Figure 1:**
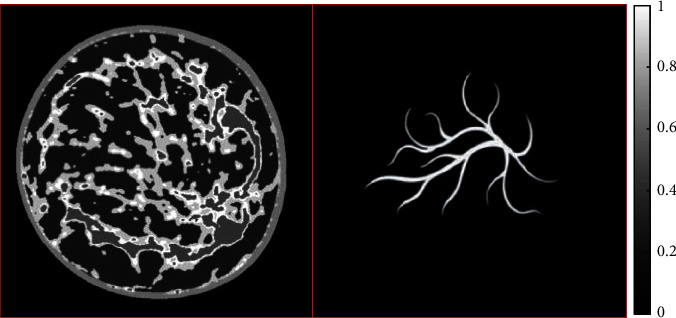
The breast phantom and the blood vessel phantom.

Figure 1 shows the breast phantom and the blood vessel phantom. The phantoms and Equation (4) are used to produce the photoacoustic signals. The size of the phantom is 32 mm × 32 mm and the resolution is 128 × 128 pixels. During the simulation experiment, the diameter of the circular scan of the ultrasonic transducer is 45 mm, and the sound velocity of the ultrasonic is 1500 m/s. At each sampling position of the ultrasound detector, 64 samples randomly selected in the (0.15, 4) MHz window are used to define the projection matrix *K*_(*m*, *n*)(*i*, *j*)_ using Equation (4). By normalizing the gray value of the phantom to (0, 1), the simulation signal is obtained by using the frequency domain projection matrix. The frequency domain measurement data can be generated by using *y*_(*m*, *n*)_ = *K*_(*m*, *n*)(*i*, *j*)_*x*_(*i*, *j*)_.

**Figure 2:**
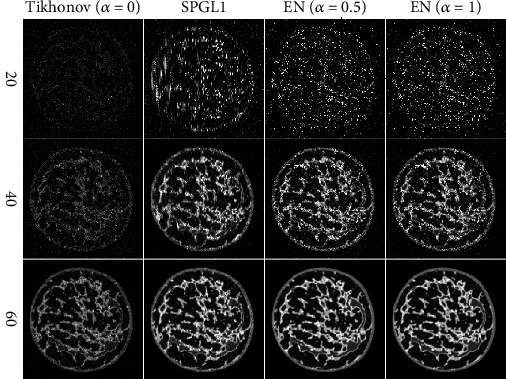
The breast phantom reconstruction results by different methods. The first to the third rows are the reconstruction results of 20, 40, and 60 views evenly distributed on the circumference, respectively. The first to fourth columns display results of the Tikhonov (*α* = 0), SPGL1, EN (*α* = 0.5), and EN (*α* = 1) individually.

Figure 2 shows the experimental results of breast phantom using these three algorithms. It can be seen that all three methods achieve good reconstruction results when using 60 position signals. Moreover, the reconstruction capability of the EN method is better than that of the Tikhonov and SPGL1 methods in the visual sense. When using 40 sampled signals, the reconstructed image of these three methods contain large noise and artifacts. In addition, Tikhonov has the worst image quality indices, followed by SPGL1. The quality of photoacoustic images reconstructed by all these three methods is poor when 20 sampled signals are used. As can be seen from Figure 2, the EN method can obtain more accurate images when the sampling number is sufficient.

**Figure 3:**
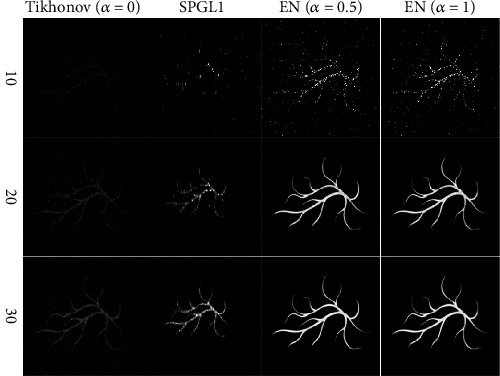
The blood vessel phantom results reconstruct by different methods. The first to the third rows are the reconstruction results of 10, 20, and 30 views that are evenly distributed on the circumference individually. The first to the fourth columns display results of the Tikhonov (*α* = 0), SPGL1, EN (*α* = 0.5), and EN (*α* = 1), respectively.

In order to verify the general applicability of the EN method, we choose the blood vessel phantom as the initial optical deposition to additionally compare these three algorithms. And this experiment has the same simulation environment as the breast phantom experiment. As can be seen from Figure 3, Tikhonov has darker and noisy reconstructed images when small amounts of signals are used. In addition, the SPGL1 method is unable to reconstruct high-quality photoacoustic images based on signals from less than 30 locations, while the EN method can reconstruct clear photoacoustic images using signals from 20 sampling locations. From the above two simulation results, it can be seen that the EN method can effectively remove noises and preserve edges.

**Figure 4:**
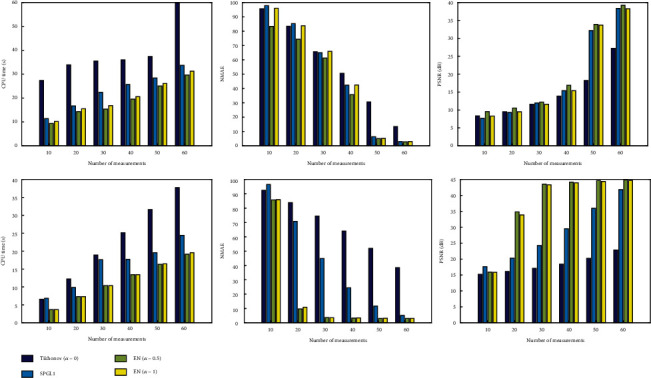
The histograms of numerical phantom results. The first and second rows represent the results of the breast phantom and the blood vessel phantom, respectively. The first to third columns are the values of evaluation index CPU time, NMAE, and PSNR as a function of the number of measurements.

As can be seen from the first column of Figure 4, the CPU time becomes larger when the number of measurements becomes larger. The running time of two phantom experiments is similar. The Tikhonov method takes the longest time to reconstruct, and the EN method has the shortest reconstruction time. From the second column of Figure 4, we can see that the larger the number of samples, the lower the NMAE. In the breast phantom experiment, 50-60 sampled signals are required to obtain a small NMAE value, while in the blood vessel phantom experiment, only 30 sampled signals are required to obtain a satisfactory NMAE value. It can be seen from Figure 4 that the Tikhonov method (*α* = 0) fail to generate smaller NMAE and larger PSNR even with 60 sampled signals. In the blood vessel phantom experiment, when the number of sampled signals is sufficient, the NMAE and PSNR obtained by the SPGL1 method are similar to those obtained by the EN method. However, when fewer signals are used, the PSNR of the SPGL1 method is much lower than that of the EN method. The above two simulations verify that the EN with *α* = 0.5 and *α* = 1 yields a higher time resolution and a higher reconstruction quality than the Tikhonov and SPGL1 methods.

### 3.2. Antinoise Ability Experiment

**Figure 5:**
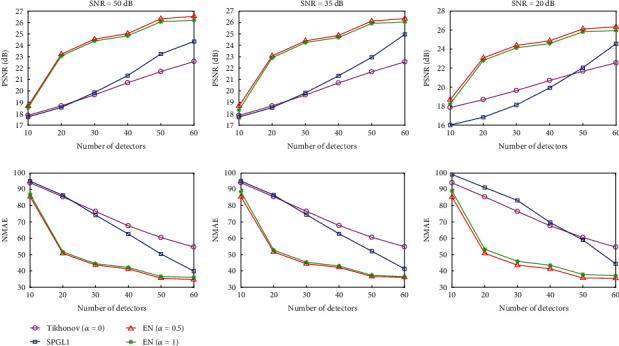
The trend of PSNR and NMAE values with increasing sampling number. The first and second rows show the trend graph of the PSNR and NMAE values of reconstructed images using signals with noise levels of SNR = 50, 35, and 20 dB, respectively.

Noise is likely to be added during photoacoustic signal acquisition. To assess the accuracy and stability of the EN methods, we added various levels of noise to the simulated data and investigate the effects of noise on the reconstructed images. A stable algorithm has higher PSNRs and smaller NMAEs. The numerical results of the PSNR and NMAE from the blood vessel phantom experiment are shown in Figure 5. As can be seen from Figure 5, the EN method achieves maximum PSNR and minimum NMAE at various noise levels. The PSNRs of the Tikhonov methods lightly drop with the increase of noise level, and the PSNRs of theSPGL1 algorithm decrease the most, while the PSNRs of the EN algorithm are essentially unchanged. The results of the antinoise ability experiments confirmed that EN is the most robust of these approaches. And EN with *α* = 0.5 has better noise robustness than EN with *α* = 1.

### 3.3. Parameter Investigation

**Figure 6:**
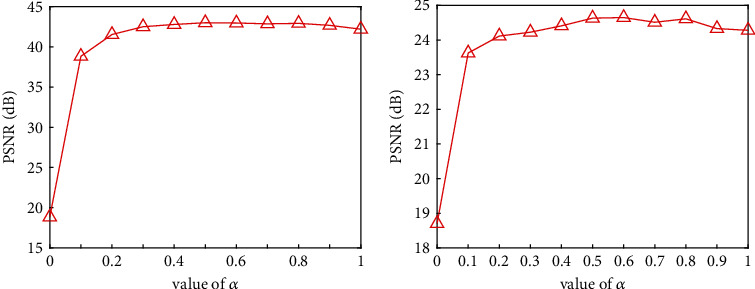
The PSNR values with different alpha and 30-view simulated data. (a) The line chart displays the quantitative results of noiseless data, and (b) the line chart shows the quantitative results of noisy observation with SNR = 10 dB.

In this subsection, we investigate the impact of the regularization parameter *α* on image quality reconstructed by the EN method. Here we select 30-view simulated data from the blood vessel phantom to further investigate the parameter settings. And the value of *α* ranges from 0 to 1. We calculate the PSNR value of the reconstructed image, and the line charts of PSNRs are shown in Figure 6. The numerical simulation experiment shows that, for noiseless data case, the PSNR value becomes larger and larger when the alpha value changes from 0 to 1, and it changes very little when the alpha value is from 0.4 to 1. Nevertheless, for noisy data case, EN with *α* = 0.5 has the maximum PSNR value, that is, has better noise robustness.

### 3.4. Tissue-Mimicking Phantom Experiment

**Figure 7:**
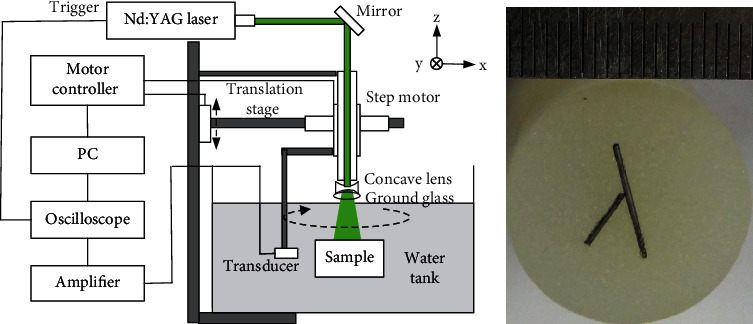
(a) The schematic of PAI system. (b) Cross-sectional view of a cylinder agar phantom containing two carbon rod absorbers.

We performed the tissue-mimicking phantom experiment to evaluate the practicability of the EN approach. In Figure 7(a), the schematic of experimental set-up is shown. A Q-switched 532 nm Nd:YAG laser with a frequency resolution of 10 Hz was applied as the light source. The input laser pulse was amplified by a concave lens, homogenized with ground glass, and then irradiated onto a sample made of agar and black carbon sticks. A 5 MHz single-element ultrasonic transducer with a diameter of 12.7 mm (V309, Panametrics) was used to receive the photoacoustic signal. The transducer and the sample are submerged in a water tank to couple the photoacoustic waves to the transducer. A stepper motor (PMC100-3) controls the transducer to rotate around the sample for sampling. The rotation radius of the transducer is 40 mm. At every sampling point, the ultrasound signal was first amplified by a Panametrics pulse amplifier and then captured and averaged 30 times by an oscilloscope (MSO4000B; Tektronix). A personal computer is used to control the stepper motors and signal acquisition [36].

**Figure 8:**
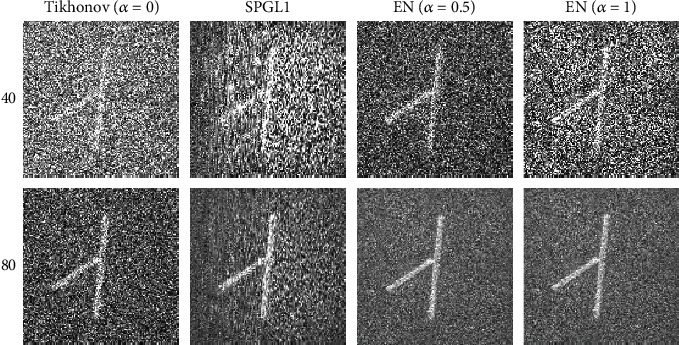
The results of agar phantom experiment. The first and second rows are the reconstruction images of carbon absorption sample from 40-view and 80-view experimental data. The first to fourth columns are the results of the Tikhonov (*α* = 0), SPGL1, EN (*α* = 0.5), and EN (*α* = 1), individually.

Figure 7(b) is the cross-sectional view of a cylinder agar phantom containing two carbon rod absorbers. The radius of the sample is 10 mm. Two carbon rods with a 0.5 mm diameter and lengths of 5 mm and 10 mm were embedded in the phantom as the optical absorbers. In the reconstruction experiment, signals at 40 and 80 positions evenly distributed on the circumference are used.  Figure 8 was reconstructed by 64 frequency samples randomly chosen inside the (0.25, 5) MHz window. And the transducer response *g*_*n*_ was restricted to certain value 1.

Figure 8 shows the results of agar phantom experiment. The images are constructed by the Tikhonov, SPGL1, and EN methods, respectively. There are too many noises in the images reconstructed by the Tikhonov and SPGL1 methods using 40-view signals, which cause all the details of the phantom to be suppressed. Although the image reconstructed by the EN method with 40-view data also has a lot of noise, the approximate outline of the phantom can be seen. The second row of Figure 8 is reconstructed from 80-view signals. When the number of sample points is sufficient, all three methods are feasible. The EN method with *α* = 0.5 achieves the best results in terms of reconstruction accuracy, visual effects, and noise robustness among the three algorithms.

## 4. Conclusion

The principal purpose of this paper was to evaluate the application of the EN method in PAI. Based on CS theory, the EN method can reconstruct photoacoustic images using a small amount of data. To evaluate the reconstruction performance of EN method, we compare it with the Tikhonov and the SPGL1 in terms of visualization and performance indicators. The reconstruction results show that the EN method provides good imaging quality; it also simultaneously has acceptable time efficiency and better robustness. And the EN with *α* = 0.5 has better noise robustness than EN with *α* = 1. Future work will focus on the biomedical applications of the EN method.

## Data Availability

The data that support the findings of this study are available from the corresponding author upon reasonable request.
